# Dissociable Self Effects for Emotion Regulation: A Study of Chinese Major Depressive Outpatients

**DOI:** 10.1155/2014/390865

**Published:** 2014-04-03

**Authors:** Xiaoxia Wang, Zhengzhi Feng, Daiquan Zhou, Xu Lei, Tongquan Liao, Li Zhang, Bing Ji, Jing Li

**Affiliations:** ^1^Department of General Psychology, College of Psychology, Third Military Medical University, Street 30, Gaotanyan of Shapingba District, Chongqing 400038, China; ^2^Radiology Department, Southwest Hospital, Third Military Medical University, Chongqing 400037, China; ^3^School of Psychology, Southwest University, Chongqing 400715, China; ^4^Xin Qiao Hospital, Third Military Medical University, Chongqing 400038, China

## Abstract

Reappraisal is an adaptive emotion regulation strategy while the role of self-perspective in reappraisal process of depressed patients is largely unknown in terms of goals (valence/arousal) and tactics (detachment/immersion). In this study, 12 depressed individuals and 15 controls were scanned with MRI during which they either attend naturally to emotional stimuli, or adopt detachment/immersion strategy. Behaviorally, no group differences in self-reported emotion regulation effectiveness were found. In addition, we observed that (1) patients were less able to downregulate amygdala activation with recruitment of more dorsal lateral prefrontal cortex (dlPFC) when adopting detachment strategy regardless of valence, and this preserved ability to regulate emotion was inversely associated with severity of symptoms; (2) patients had deficits in upregulating amygdala activation when adopting immersion strategy, with less inferior frontal gyrus (IFG) activation and strengthening coupling of dlPFC and ventral medial prefrontal cortex (vmPFC) with amygdala; (3) comparison between groups yielded that patients showed stronger vmPFC activation under either self-detached or self-immersed condition. In conclusion, impaired modulatory effects of amygdala in depressed patients are compensated with strengthening cognitive control resources, with dissociable effects for different self-perspectives in reappraisal. These results may help clarify the role of self-perspective underlying reappraisal in major depression.

## 1. Introduction

According to the fourth edition of Diagnostic and Statistical Manual of Mental Disorders (DSM-IV), anhedonia and/or a lingering low mood are the defining characteristics of major depressive disorder (MDD). A body of evidence implies a trait-like role of maladaptive emotion regulation strategies in MDD vulnerability [1], among which the strategy of reappraisal has received the most extensive attention. In clinical settings, reappraisal plays a pivotal role in cognitive behavioral therapy (CBT) interventions [2] that predicts resilience in depressed patients [3]. Converging neuroimaging evidence indicates that reappraisal engages the lateral and medial sectors of the dorsal/ventral prefrontal cortex and subcortical structures such as amygdala. Notably, these structures are also foci of brain network dysfunction in the neurological models of depression [4, 5] which propose that attenuated top-down cognitive control networks are companied with unrestrained activation in emotional regions (i.e., amygdala) [6–9]. Reappraisal may involve the utilization of cognitive control to regulate semantic representations of affective stimuli which in turn attenuate amygdala reactivity [10]. Moreover, reappraisal is generally viewed as an adaptive emotion regulation strategy which is dysfunctional in depression with less frequency of daily use [11] as well as abnormal neural activation patterns [9, 12, 13]. Therefore, reappraisal may be a promising target for disclosing the vulnerable characteristics of depression.

Operationally, reappraisal refers to a combination of approaches that require generation, maintenance, coordination of top-down cognitive reinterpretation, and bottom-up appraisal of affective events and monitoring them in working memory over time [14]. Reappraisal strategies can vary in their goals (what is regulated) and tactics (how is emotion regulated), leading to multiple variants of experiment paradigm [15].

The circumplex model of affect suggests that all emotions can be distinguished in terms of varying levels of valence and arousal, with two distinct neural systems mediating the representation of affective states. It is addressed that common as well as distinct neural substrates underlie the regulation of different valences of emotion [16]. Primarily, there could be two reappraisal goals (what people want to achieve) that effectively regulate negative emotion: to upregulate positive emotion and downregulate negative emotion [15]. Positive emotion can be used to counter negative emotion in psychologically resilient individuals [17], spiral upward toward positive reappraisals, and transform negative affect into positive affect, leading to optimal functioning [18]. However, most studies of reappraisal in major depression focus on decreasing negative emotion, possibly due to excessive negative affect prevalent in MDD. To date only two studies to our knowledge explore positive emotion regulation in MDD, and they adopt different reappraisal working definitions. One study reports that depressed individuals fail to sustain activation in neural circuits underlying positive affect [19]. The other study finds no significant differences in downregulating positive affect by reappraisal between normal controls and MDD [12]. Neither study simultaneously investigates both processes, making it open to question whether only one or both processes are impaired. Positive and negative affect may facilitate the use of different sources of information, in terms of relation between self and situation [20]. Nonetheless, restriction to one valence makes it difficult to generalize the conclusion about reappraisal in common emotional state.

Another critical question remains to be answered is what is regulated. Reappraisal can be self- or situation-oriented [21]. The former focuses on reinterpreting the personal meaning of the emotional object to make it more or less self-relevant, while the latter focuses on reinterpreting the consequence or the reality of emotional stimuli without changing one's relationship to the stimuli [22]. In some studies, self-oriented reappraisal with decreasing affect as the regulating goal is also called detached reappraisal [23–25] or distancing reappraisal [15], which is efficient for emotion regulation [26]. In these studies, reappraisal is conceptualized as taking an objective or third-person perspective upon the emotional stimuli/situation. Reappraisal had been the target of clinical research on stress coping and CBT long before the conduction of laboratory experimental research on reappraisal as a form of emotion regulation. In line with this tradition, other researchers with social and clinical background deem self-distancing as a form of self-reflection and introduce psychological distance to distinguish adaptive versus maladaptive self-reflection [26–29]. Recent evidence has linked effectiveness of self-distancing to adaptive behavioral outcomes [27]. It is reported that depressed individuals can benefit from analyzing negative emotion events from a self-distancing perspective [28]. Moreover, evaluative rather than experiential self-referential processing is inherent in depression [30]. However, the modulatory effect of self-focused processing on reappraisal has been deemphasized. One feasible approach is to validate the role of self-focused processing in reappraisal and to manipulate reappraisal strategies along this dimension.

The purpose of this study was to examine the neural mechanisms of self-related reappraisal in Chinese MDD outpatients. Block designs were employed to avoid naturally declining emotion processes when watching emotionally arousing pictures. Emotion control areas such as dorsal lateral prefrontal cortex (dlPFC) and ventral medial prefrontal cortex (vmPFC) were selected as regions of interest since these two areas seemed to be involved in pathogenesis of depression and influence the expression of depressive symptoms [4, 31]. Amygdala was examined because it could act as a neural proxy for changes in emotion induction [22]. We hypothesized that (1) self-related neural networks underlying reappraisal would be differentially activated in depressed patients versus controls; (2) major depression would show abnormal neural activations underlying self-related reappraisal of affect.

## 2. Methods

### 2.1. Participants

Twelve unmedicated major depressed outpatients and 15 normal controls were recruited. The patients were diagnosed through a structured clinical interview according to DSM-IV. For depressed patients, an inclusion criterion with current depressed episode was adopted, according to the DSM-IV. All patients were assessed with SDS, BDI, and HAMD before participating into experiment, with mild to moderate depression symptoms (HAMD ≥ 18; BDI ≥ 14; SDS ≥ 35). For healthy controls,semi-standard interviews were conducted and assessed with SDS and BDI, with no current depressed mood (BDI < 4 or SDS < 50). For both groups, exclusion criteria were history of neurological disease or presence of axis I psychiatric disorders, psychiatric medication use within the last two weeks, or implanted cardiac or ferrous metal devices. The patients and normal controls were also assessed with BDI/SDS and the patients with HAMD (24-item version) additionally. Statistically significant differences were found in BDI and SDS scores (Table 1). A written informed consent was obtained from all subjects before experiment. This study was approved by the Ethics Committee of the Third Military Medical University.

### 2.2. Experimental Procedure

The participants were trained on a computer during a previous session to get familiar with the reappraisal strategies they were to use during the scan. They were instructed to either attend to the visual stimuli or reappraise (self-detached versus self-immersed) their emotion reactions to each picture. Tasks were performed in three consecutive sessions after acquisition of structural images. Emotional stimuli were selected from the International Affective Pictures System (IAPS) [32] and matched for content of scenes and people as well as valence and arousal for each condition (mean valence (*V*) and arousal (*A*): neutral/attend, *V* = 5.06, *A* = 2.74; positive/attend, *V* = 7.17, *A* = 5.45; negative/attend, *V* = 2.34, *A* = 5.60; positive/detached, *V* = 7.26, *A* = 5.69; negative/detached, *V* = 2.69, *A* = 5.82; positive/immersed, *V* = 6.94, *A* = 5.30; negative/immersed, *V* = 2.42, *A* = 5.09). One-way ANOVA for stimuli in each session resulted in insignificant differences in arousal when taking valence as a factor (all *P*s > 0.05). Twelve pictures were used for each valence under each condition and one more neutral picture for the start of each condition. The neutral picture was eliminated from MRI data analysis afterwards to prevent from signal drift.

**Figure 1:**
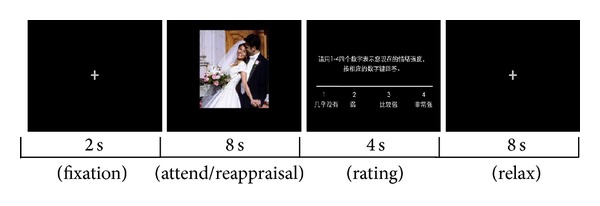
Sequence of a trials in attend/reappraisal task. Instructions were given prior to each block.

For the attend condition (session 1), subjects should simply view the picture without taking efforts to alter their emotion; for the self-immersed conditions (session 2), subjects should perceive each picture as real and engage themselves in the situation depicted, by imagining themselves or a loved one in the scene; for the self-detached conditions (session 3), subjects should view the situation as fake or unreal and detach themselves from the situation. The attend condition was set as the control condition. Participants were told not to close their eyes or direct eyes away from the pictures during each trial and be able to relax during the break after each trial (Figure 1).

During the scanning, stimuli were projected onto a screen, reflected by a mirror in front of the subjects. The task was performed in three consecutive sessions (“maintain,” “detach,” and “immerse”), in the order of the last two sequentially counterbalanced across all subjects. The instruction for each condition was given at the beginning of each block. Each trial consisted of four components: fixation, induction or regulation, rating, and rest. A fixation cross was displayed for 2 s, and then an IAPS picture appeared for 8 s during which subjects either simply viewed or reappraised the picture, followed by an affect rating screen (1 = no intensity to 4 = very intense), and a black blank screen was shown for 8 s for relaxation. A four-point scale was chosen because it forces the subjects to make emotional judgments and was proved to be reliable for emotion discrimination in a previous study [33]. Affect ratings were collected using a two-button response box held in each hand. After experiment, all the subjects were inquired to confirm the effectiveness of emotion regulation.

### 2.3. MRI Data Acquisition

MRI data were collected on a Siemens 3T Allegra MRI scanner. A high-resolution T1-weighted 3D image (T1WI) was acquired, with slice thickness = 4 mm, field of view (FOV) = 240 × 240 × 240 mm^3^, and matrix =256 × 256 × 256. Functional images were obtained from 30 gradient-echo T2*-weighted slices (slice thickness = 4 mm) per volume. A single shot gradient-recalled echo-echo planar imaging (SS-GRE-EPI) sequence was used with a time repetition of 2000 ms, a flip angle of 90°, time echo of 30 ms, FOV of 240 × 240 mm^2^, matrix of 64 × 64, slice thickness of 4 mm, and slice interval of 0.8 mm. For coregistration, 176 sagittal whole-brain scans were collected by 3-D magnetization-prepared rapid gradient-echo imaging (MPRAGE), with TR = 1970 ms, TE = 3.93 ms, a flip angle = 15°, slice thickness = 1.70 mm, slice interval = 0.85 mm, FOV = 250 × 250 mm^2^, and a matrix = 448 × 512.

### 2.4. Data Analysis

#### 2.4.1. Self-Report Data

The emotional state ratings during the experiment were analyzed with PASW (Version 19, SPSS Inc., Chicago, IL, USA). A two-way ANOVA was conducted to analyze the effect of the emotional picture presentation (negative, neutral, positive) on emotional state in the viewing condition. A 2 × 3 × 2 repeated-measures ANOVA including the factors group (MDD, HC), valence (negative, positive), and condition (attend, self-detachment, self-immersion) was calculated to illuminate the effects of regulation on emotional state. The neutral condition was neglected for the second analysis as there was no neutral picture in the reappraisal condition.

#### 2.4.2. Functional MRI Data

Data were preprocessed and statistically analyzed with SPM 8 (http://www.fil.ion.ucl.ac.uk/spm/software/spm8/) and Matlab 7.8.0 (Math Works, Natick, MA). The preprocessing included realignment, spatial normalization, and spatial smoothing (8 mm).

*GLM Analysis*. The first level analysis consisted of seven regressors (attend neutral, attend positive, attend negative, decrease positive, decrease negative, increase positive, and increase negative) modeled with a duration of 8 seconds convolved with the hemodynamic response function. A high-pass filter was applied and six head motion parameters were included as residuals. In a second level analysis, we conducted a repeated measures general linear model (GLM) with emotion and reappraisal as within-subjects factors and group as a between-subjects factor. Post hoc *t* tests were then performed to examine contrasts between factors with significant main effects and interactions. Significant difference of statistical maps for whole brain analysis was set at *P* < 0.05, corrected for multiple comparisons using cluster-size thresholding (54 voxels^2^) based on Monte Carlo simulation. 

We then performed region of interest (ROI) analyses upon a priori region of interest implicated in emotion reactivity and regulation (bilateral dlPFC, vmPFC, and amygdala). If vmPFC and dlPFC are critical neural substrates for pathogenesis of depression, then damage to either area should affect the expression of depressive symptoms. We used anatomical masks based on the Talairach daemon database, defined by WFU Pick atlas software (version 3.0; ANSIR Laboratory, WFU School of Medicine, Winston-Salem, NorthCarolina), and set the threshold at *P* < 0.05 with an extent threshold of 5 voxels [34]. As to thresholding, the incorporation of extent threshold into *P* value effectively achieved equivalent correction for multiple comparisons [35]. ROI time courses were extracted within anatomically defined ROIs by generating the first eigenvariate of 8 mm around the peak voxels using a Matlab package REX (Response Exploration) [36]. Eigenvariates were extracted and global-scaled to produce a time series of functional data in units of percent signal change referenced to the SPM default intracerebral mean of 100.

*Psychophysiological Interaction Analysis*. This analysis was performed to identify the brain regions that produce a downregulating effect on the amygdala during emotion regulation. A 10 mm seed region around the peak activation in bilateral amygdala was identified when we contrasted reappraisal and attends condition for each valence between two groups. Time series were extracted for each subject as the first regressor (physiological variable). The second regressor represented psychological variable (condition parameter). The regressor of interest was the interaction between the physiological variable and psychological variable, created from product time series of VOI and the condition parameter. We then created subtraction contrast between conditions of interests, and all individual contrast images were included into a second-level group random-effects analysis, in which task-dependent effects were investigated using a two-sample *t* test for two groups. Significant activations exhibiting PPI-related amygdala coupling were identified with a threshold *P* < 0.001 (uncorrected).

## 3. Results

### 3.1. Behavioral Results

**Figure 2:**
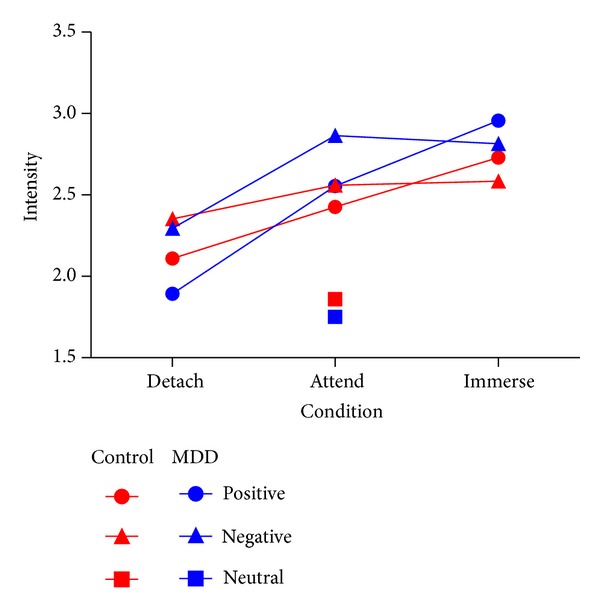
Means and standard deviations of intensity ratings after affect processing during the scan.

A one-way ANOVA was computed to analyze the effect of picture type (positive, neutral, negative) on induced emotional reactivity during the attending task. A 2 × 3 × 2 repeated-measures ANOVA was also conducted on factors including group (MDD, normal), condition (attend, self-detachment, self-immersion), and emotion (positive, negative) to examine the effects of cognitive reappraisal on emotional induction (Figure 2).

*Emotion Reactivity*. We observed a significant main effect of emotion (*F* (2, 22) = 44.9, *P* < 0.001) during the attending task. Pairwise comparisons showed that negative and positive trials differed from neutral trials (*P* < 0.001). There was no difference between MDD patients and normal controls (*P* > 0.455).

*Emotion Regulation*. The emotional state ratings yielded a significant main effect of condition (*F* (2, 21) = 15.620 *P* = 0.000, partial *η* ^2^ = 0.415) and emotion (*F* (1, 22) = 11.355, *P* = 0.003, partial *η* ^2^ = 0.340) and a significant interaction between condition and emotion (*F* (2, 21) = 14.215, *P* = 0.000, partial *η* ^2^ = 0.575). Either group main effect or group-related interactions were insignificant (*P* > 0.05). Post hoc contrasts indicated that emotional intensity was significantly regulated via self-detachment (*P* = 0.001) and self-immersion (*P* = 0.007), compared to the viewing condition and to each other (*P* = 0.001). These results suggested that both groups successfully regulated emotions without significant group differences.

### 3.2. Functional MRI Results

#### 3.2.1. Activation Analysis

*Factor Analysis*. We used a full factorial design ANOVA in the second-level group random-effects analysis. There was no significant group × reappraisal × emotion interaction. Group × reappraisal interactions were found in right medial temporal gyrus (MTG), left inferior frontal gyrus (IFG), left superior temporal gyrus (STG), right lingual gyrus, left thalamus, left amygdala, and left insula.

**Figure 3:**
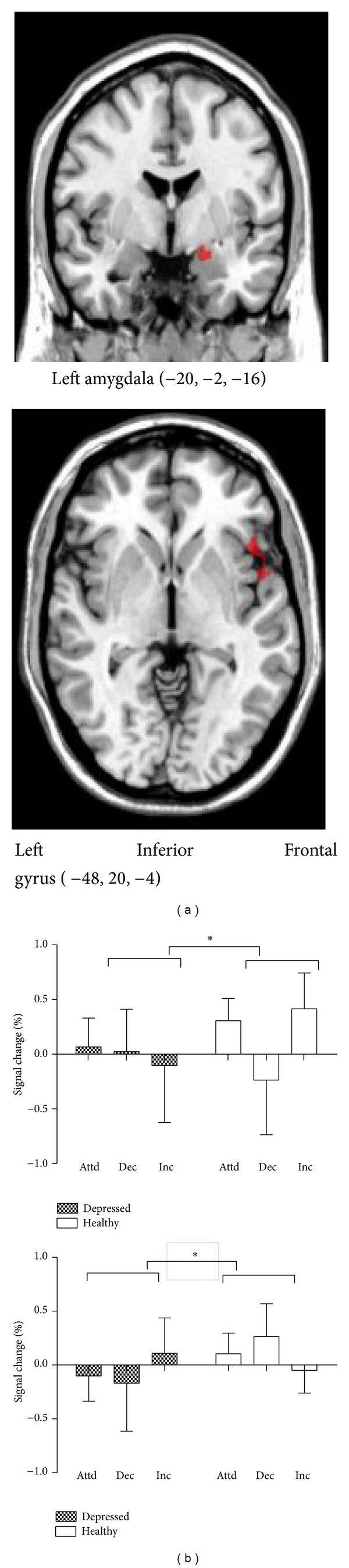
(a) Group × reappraisal interaction: regions activated in “attend,” “detach,” and “immerse” conditions in both groups. (b) Percent signal change for the following contrasts: “attend/emotional,” “detach/emotional,” and “immerse/emotional”. Note: attd = attend; dec = detach; inc = immerse.

Regarding priori regions, we found activations in left inferior frontal gyrus (IFG) (109 voxels in left IFG, peak at (−48, 20, −4) *t* = 3.07, *P* < 0.01) and left amygdala (40 voxels in left amygdala, peak at (−20, −2, −16) *t* = 2.42, *P* < 0.01). A post hoc t-contrast revealed that the group × reappraisal interaction was explained by hypoactivation of left amygdala in “immerse minus attend” contrast, hypoactivation of left IFG in “detach minus attend” contrast, and hyperactivation of left IFG in “immerse minus attend” contrast. Percent signal changes in each ROI were extracted and entered into SPSS for group × reappraisal two-way ANOVA, resulting in similar activation-deactivation pattern (Figure 3). Because we did not observe significant three-way interaction between group, reappraisal, and emotion, we did not take emotional valence into account. We also observed main effects for group, emotion, and reappraisal.

*Region of Interest Analysis*. To identify the neural correlates of regulatory effects on amygdala activity due to reappraisal, we performed ROI analysis on bilateral amygdala using one-sample *t* test in the control group. For detach effects, we observed decreased amygdala activity during “attend/positive > detach/positive” contrast (9 voxels in left amygdala, peak at (−18, −4, −18) *t* = 2.17, *P* < 0.05) and decreased amygdala activity during “attend/negative > detach/negative” contrast (6 voxels in left amygdala, peak at (−20, −10, −10) *t* = 2.57, *P* < 0.05). For immerse effects, we observed increased amygdala activity during “immerse/positive > attend/positive” contrast (30 voxels in right amygdala, peak at (22, −6, −18) *t* = 3.56, *P* < 0.005) and during “immerse/negative > attend/negative” contrast (11 voxels in left amygdala, peak at (−18, −4, −26) *t* = 2.77, *P* < 0.01; 44 voxels in right amygdala, peak at (20, −4, −26) *t* = 4.57, *P* < 0.001). We performed similar test on the patient group and found no regulatory effects of amygdala in any individual contrast.

We then performed a two-sample *t* test for anatomical ROIs between MDD and the control group. The investigation of all contrasts of interest did not include bilateral amygdala, as no regulation effects of amygdala were found in patients and thus incomparable between groups. For “detach/positive > attend/positive” condition, left vmPFC showed greater activation for patients than for controls (15 voxels in left vmPFC, peak at (−4, 54, −10) *t* = 2.61, *P* < 0.01). For “detach/negative > attend/negative” condition, right dlPFC and vmPFC were more active in patients than in controls (8 voxels in right dlPFC, peak at (14, 40, 20) *t* = 1.99, *P* < 0.05; 37 voxels in right vmPFC, peak at (38, 34, −14) *t* = 3.71, *P* < 0.001; 18 voxels in right vmPFC, peak at (6, 52, −10) *t* = 3.13, *P* < 0.005; 53 voxels in right vmPFC, peak at (10, 34, 20) *t* = 2.22, *P* < 0.05). For “immerse/positive > attend/positive” condition, left vmPFC was more active in patients than in controls, and right vmPFC was more active in controls than in patients (20 voxels in left vmPFC, peak at (−4, 56, −6) *t* = 2.23, *P* < 0.05; 7 voxels in right vmPFC, peak at (24, 34, −12) *t* = 0.02, *P* < 0.05). For “immerse/negative > attend/negative” condition, right dlPFC and bilateral vmPFC showed greater activation for patients than for controls (12 voxels in right dlPFC, peak at (12, 40, 18) *t* = 2.26, *P* < 0.05; 12 voxels in left vmPFC, peak at (−4, 46, 12) *t* = 1.84, *P* < 0.05; 66 voxels in right vmPFC, peak at (10, 38, 18) *t* = 2.52, *P* < 0.01).

**Figure 4:**
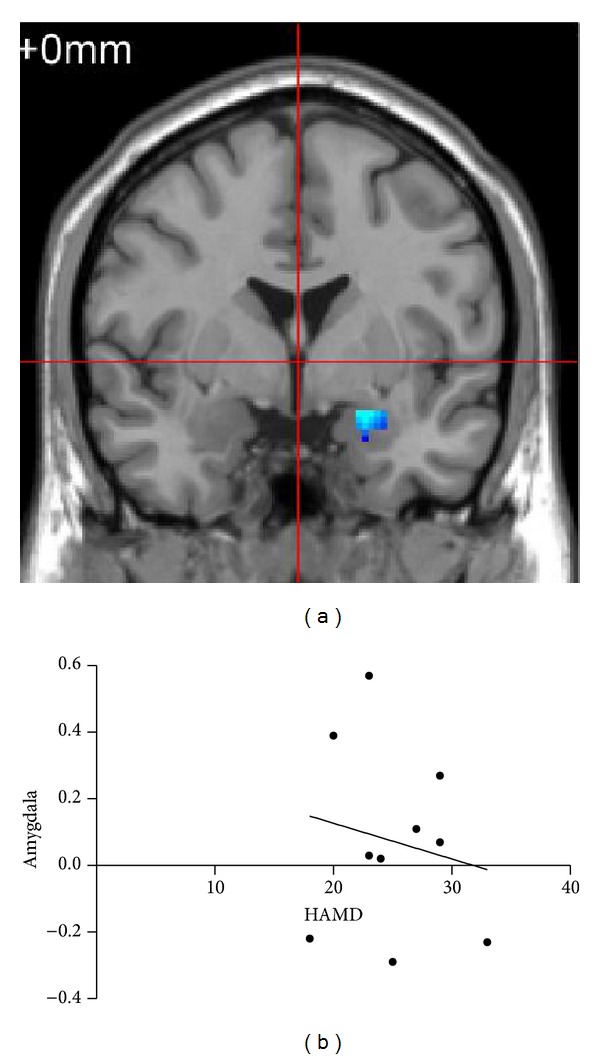
(a) Decreased activation in the left amygdala for patients during detachment of positive emotion. (b) The signal change of amygdala between the detachment and view conditions correlated negatively with HAMD scores (*r* = −0.608, *P* = 0.036).

In patients, a regression analysis revealed that during detachment of positive emotion, downregulation of left amygdala negatively correlated with HAMD scores (*r* = −0.608,  *P* = 0.036, two-tailed), suggesting that the more severe the depression symptom is, the less effective the downregulation of amygdala will be (Figure 4).

#### 3.2.2. Psychophysiological Interaction Analysis

We are specifically interested in amygdala-cortical interactions during reappraisal. The PPI analysis revealed that compared to healthy controls, patients showed significantly enhanced coactivation of left amygdala with right dlPFC (MFG), right vmPFC (anterior cingulate), and right inferior parietal lobule (IPL) (Table 2).

## 4. Discussions

The current study extends previous findings about neural correlates of reappraisal in MDD along a self-relatedness dimension and confirms the hypotheses that self-relatedness may differentially modulate neural circuits underlying reappraisal for MDD versus normal group, demonstrating inflexible amygdala reactivity and strengthening frontolimbic connection in MDD. Since these neural circuits are involved in the pathology of depression [37], the current study may provide further evidence on how this abnormal functional connectivity pattern translates into emotion dysregulation in depression.

Behaviorally, IAPS stimuli significantly induced emotion in both groups (*P* < 0.05). In addition, both groups were equally effective in using reappraisal strategies to up- and downregulate emotions. Neurally, within-group region-of-interest analysis indicates the regulation effects of reappraisal on amygdala in controls, consistent with previous studies [38–40], suggesting neural correlates as more sensitive indices of regulation outcome. It is noteworthy that this pattern of amygdala reactivity was not observed in the MDD group. Previous studies are inconclusive regarding whether the ability to regulate amygdala activation is deteriorated in MDD. One possible explanation for the discrepancy between studies is the heterogeneity of regulation goals, which use self-ratings of valence and/or arousal as behavioral measures. Some studies report that controls and depressed individuals show comparable amygdala responses to emotional stimuli in “detach > attend” contrast [6, 12, 41]. However, Johnstone's study does not observe “decrease-attend” reappraisal effect on amygdala activation in either controls or depressed patients [13]. Empirical evidence indicates that amygdala belongs to both valence and arousal networks [42]. In particular, amygdala is sensitive to arousal when valence remains unchanged and is dormant to valence changes when arousal remains constant [43]. Thus, regulation goals putatively cause distinct amygdala response patterns which may be ignored in previous studies.

Interestingly, Dillon's study using valence ratings as emotion responses revealed comparable regulation outcome between groups in increasing emotion, which differs from current study. Moreover, post hoc contrasts of repeated-measures ANOVA demonstrate that the left amygdala of depressed patients is less activated when immersion strategy is adopted regardless of valence (see in Figure 3). This blunted amygdala activity is aligned with an emotion context insensitivity (ECI) view which depicted flattened emotional responses typical of MDD [44]. Accordingly, event-related potential (ERP) study also addressed diminished brain responses during sustained processing of positive information [45]. Our study was comparable to previous study showing that depression fails to maintain positive emotions, with a different brain foci of positive emotion processing (ventral striatum) [19]. The disparity between studies is comprehensible in that our study adopted emotional arousal as an indicator of regulation outcome, while in Heller's study, emotional valence was adopted. Furthermore, less amygdala activity during immersion is also consistent with its role in representation with arousal [46].

In contrast to healthy controls, depressed individuals exhibit diminished activation in left IFG when detachment strategy is adopted and enhanced activation in left IFG when immersion technique is adopted (see in Figure 3). IFG (BA47) is implicated in inhibitory control in emotional as well as cognitive domains [47]. This abnormal control-related activation can be viewed as a functionally compensatory process in response to behavioral deficits, in spite of preserved emotion regulation behavioral measures for MDD. Altogether, the present results indicate compromised functioning of MDD in resistance to affective interference and inhibiting spontaneous emotional responses and supported the assumption that MDD emotion dysregulation is spanning negative as well as positive affect.

Our study observes that in depressed patients, left vmPFC is more strongly recruited for self-detachment from positive affect, while right vmPFC is more strongly activated for negative affect. Observations are similar concerning self-immersion strategy. Medial prefrontal cortex (MPFC) is central to neural models of depression [48]. In resting state, MDD patients exhibit overall increase in ventromedial PFC activation from pre- to posttreatment [49]. Task-related vmPFC activation is observed in self-oriented reappraisal [21] or when negative emotion is decreased [50]. VmPFC may be responsible for preattentively tagging both explicit and implicit incoming information as self-relevant [51, 52], representing the “Me” mode of self-reflection [53]. Our observations suggest that under both self-detached and self-immersed conditions, depressed patients show an excessive mode of self-relevance detection. This may in turn orient ongoing dorsal-ventral PFC connectivity, in line with positive correlation of elevations of negative affect with brain activations in medial PFC [54]. This self-focused cognitive tendency may constitute a basis for rumination style preferentially prevalent in depression [55].

We further assume that comparisons within each valence might suggest a possibility for hemispheric asymmetry of prefrontal cortex in MDD: left vmPFC is more involved in positive affect reappraisal and right vmPFC more in negative affect reappraisal. However, more conclusive results may rely on ANOVA including “hemisphere” as one of the factors, which is not considered in this paper because SPM 8 does not allow four-way ANOVA. It is in line with the consensus that positive/negative emotion is parallel to approach/avoidance motivation system [56], which shows evidence of hemispheric specialization in MFG [57].

The PPI analysis exhibited a strengthened coupling between left amygdala and right prefrontal cortex (including dlPFC and vmPFC) and right IPL in MDD when increasing emotion. In comparison, two-sample *t* test of whole brain analysis showed enhanced activations of dlPFC and vmPFC in MDD, together with over-reactivity of limbic-paralimbic (insula, parahippocampal gyrus) and subcortical (thalamus) structures. DlPFC (BA9, 46) is seen as the neural substrate for emotion regulation [58] and is more preferentially engaged in negative than positive stimuli in depressed patients [59]. Furthermore, dlPFC is responsible for recruiting attention control resources in reappraisal [60] and is more engaged with increasing cognitive load [61]. This result further supports our proposal that MDD may have deficits in upregulating emotion accompanied with heightened inhibitory control. Right IPL is involved in downregulation of emotion through detachment in healthy and depressed group [12], while the absence of detachment-related coactivation with amygdala for MDD is not discussed. Our findings further address a strengthened coupling of right IPL and amygdala for MDD during enhancing emotion. IPL is associated with cognitive inhibition [62], taking the perspectives of others in processing visual information [63] or orienting away from salient stimuli [64]. Collectively, from detachment to immersion, impaired functional connectivity between IPL and amygdala emphasize that depressed patients may have deficits in reappraisal which is essential for regulating emotion in both directions.

A wealth of data suggests that amygdala, insula, and anterior temporal pole are responsible for separately mediating the cognitive, physiological, and experiential aspects of emotional responses, respectively [41]. The insula serves as a strategic neural node in the appraisal of emotional responses [65]. We observed strengthened task-related coupling of amygdala with insula; amygdala response under self-immersion condition was less active in patients than in controls, implying the attenuated ability of MDD to flexibly intensify emotion reactivity. This also confirms MDD's positive emotion dysregulation assumption.

In conclusion, depressed individuals tend to rely more on cognitive control brain networks and enhanced functional coupling between left amygdala and right prefrontal cortex when using reappraisal strategy accompanied by unrestrained self-related affective processing, which applies for both valence of emotion.

### 4.1. Implications and Limitations

There may be several clinical implications of our findings. First, group differences in the ability to regulate emotion may represent a sign of vulnerability to depressed mood and depressive disorders under stress [66]. Thus, focusing on affect regulation provides a ready bridge to intervention research [67]. Second, the abnormal prefrontal activation in response to an emotion-eliciting context may be second, since the amount of downregulation of positive emotion changes with depression severity, which does not necessarily disappear with symptom recovery or medication. The underlying neurobiological changes could be used to monitor the responsiveness of patients and the effectiveness of psychotherapy.

Until now, the effect of cross-culture variability on reappraisal strategies remains largely unknown. Our study may provide preliminary insights into relevant research. The linguistic nature of emotion regulation strategies may vary among different cultures. As previous findings suggest, emotion-regulation strategies may contribute to differences in emotional experience across western and east Asian cultures [68]. Hence, replication and comparative study between ethnic groups should be targeted.

The present research has several limitations. First, stimuli of personal relevance may need to be adopted (such as autobiographical experiences) in further study. Reappraisal involves momentary relevance and meaning of current stimuli which may vary among individuals, and subject-specific stimuli according to personal relevance may maintain stimulus consistency within individuals [69]. Second, this study does not allow making causal conclusions of reciprocal connection between brain regions. This issue could be further investigated with methods such as dynamic causal modeling (DCM) or granger causal modeling (GCM) for more confirmative conclusions about causal relations or in combination with ERP technique to keep track of ongoing mental processes on finer time scale. Third, the sample size of this study is small, but the findings are well aligned with previous studies of emotion regulation. The findings in this study can serve as a basis for further investigation with a larger sample size and stronger statistical power [70].

## Figures and Tables

**Table 1:** Demographic and clinical data of MDD and normal control groups.

Measure	MDD	Control	Sig.
	Mean/SD (*n* = 12)	Mean/SD (*n* = 15)	
Gender ratio (male/female)	5/7	7/8	0.55
Age	29.50 (8.46)	25.80 (5.89)	0.07
Years of education	14.00 (3.77)	14.80 (2.83)	0.53
Handedness	right (12)	right (15)	—
BDI	26.17 (12.65)	4.27 (4.23)	0.00
SDS	64.08 (12.60)	36.54 (5.74)	0.00
HAMD	25.23 (4.97)		—

Note: BDI: beck depression inventory; SDS: self-rating depression scale. Both groups were matched for age, sex ratio, and years of education.

**Table 2:** PPI analysis of left amygdala seed for immersion/emotional condition in both groups.

Region of coactivation	Side	BA	Talairach coordinates			*Z* score
			*x*	*y*	*z*	
Control > MDD						
Middle temporal gyrus	L	39	−52	−70	30	2.18
Middle temporal gyrus	R	21	60	−18	−24	3.26
Superior temporal gyrus	R	38	44	18	−34	3.05
Medial frontal gyrus	L		−12	56	8	2.89
Control < MDD						
Insula	L		−40	−10	6	5.01
Middle frontal gyrus	R	9	42	42	32	2.75
Superior frontal gyrus	R		26	54	32	2.46
Inferior parietal lobule	R	40	42	−32	62	2.34
Precuneus	R	7	18	−36	58	2.19
Anterior cingulate	R	32	10	36	−10	2.16
Precuneus	L		−6	−44	50	1.97

## Abbreviations

dlPFC: Dorsal lateral prefrontal cortex
IFG: Inferior frontal gyrus
vmPFC: Ventral medial prefrontal cortex
DSM-IV: Fourth edition of Diagnostic and Statistical Manual of Mental Disorders
MDD: Major depressive disorder
CBT: Cognitive behavioral therapy
BDI: Beck depression inventory
SDS: Self-rating depression scale
HAMD: Hamilton rating scale for depression
IAPS: International affective pictures system
FOV: Field of view
TE: Time echo
TR: Time repetition
ROI: Region of interests
VOI: Volume of interests
PPI: Psychophysiological interaction.
